# Epidemiological Investigation and Partial NS5 Sequence Analysis of Duck Tembusu Virus in Several Regions of China in 2024

**DOI:** 10.3390/v18040400

**Published:** 2026-03-24

**Authors:** Wenxin Li, Yang Li, Qingling Ren, Yang Wang, Chengjie Cai, Ying Wang, Xiaohui Yu, Yixin Wang, Hualei Liu

**Affiliations:** 1 China Animal Health and Epidemiology Center, Qingdao 266032, China; 13644523209@163.com (W.L.); liyang@cahec.cn (Y.L.); 15032513704@163.com (Y.W.); ccj17865687870@163.com (C.C.); 19806102120@163.com (Y.W.); yuxiaohui@cahec.cn (X.Y.); 2 College of Animal Medicine, Shandong Agricultural University, Tai’an 271018, China; rqingling@163.com

**Keywords:** duck Tembusu virus, epidemiological investigation, NS5, sequencing analysis

## Abstract

In order to investigate the prevalence of duck Tembusu virus (DTMUV) in several regions of China, this study conducted an epidemiological survey on 2674 avian throat swab samples (including chickens, ducks, geese, and pigeons) collected from seven provincial-level administrative regions in China in 2024. Following RT-qPCR testing, 198 positive samples were identified, demonstrating an overall positivity rate of 7.40% (198/2674) across the seven provinces included in the study. Subsequent virus isolation using BHK-21 cells led to successful isolation in 17 cases. Additionally, genetic evolution analysis of the partial NS5 gene was carried out on these 17 isolates through RT-PCR amplification and sequencing. The data analysis indicated that Guangdong Province had the highest positive detection rate, reaching 22.40% (86/384), followed by Henan at 12.24% (47/384). Among infected hosts, geese were primarily affected by DTMUV, with a positivity rate of 40.76% (97/238). The prevailing subgroup of DTMUV in circulation in China is subgroup 3.2. Farmer’s markets, wholesale markets, slaughterhouses, and poultry farms all showed evidence of DTMUV presence, indicating widespread contamination across diverse locations. This study examines the distribution, genetics, and phylogenetic features of DTMUV in China, which will enhance our comprehension of the epidemiological landscape of DTMUV in China.

## 1. Introduction

Duck Tembusu virus (DTMUV) belongs to the *Ntaya* virus group within the genus *Flavivirus* of the family *Flaviviridae*, which is the largest genus in the family with more than 70 viruses, including Japanese encephalitis virus (JEV), dengue virus (DENV), West Nile virus (WNV), Zika virus (ZIKV), and other zoonotic viruses [1,2]. DTMUV is a mosquito-borne arbovirus that primarily circulates among mosquitoes. It was first identified in Culex mosquitoes in Malaysia in 1955 and named after the village of Tembusu in the country [3]. Although its pathogenicity in poultry was not initially recognized, sporadic cases of infection were later reported during epidemiological investigations in Southeast Asia (SEA) in 1970 [4]. A retrospective study published in 2018 confirmed the presence of DTMUV in duck populations in Thailand at that time by analyzing clinical samples from diseased ducks collected in 2007 [5]. Pathological and virological analyses identified viral infection, and phylogenetic analysis showed that the 2007 Thai DTMUV strains belonged to genetic cluster 1, which differs genetically from the strains that later circulated in China and Thailand (mainly belonging to genetic cluster 2). This indicates that the virus had silently circulated in Southeast Asia before the first DTMUV outbreak was reported in China in 2010 [6]. Infected ducks displayed symptoms such as high fever, decreased appetite, neurological manifestations such as ataxia and limb paralysis [7], and a significant reduction in egg production, known as egg drop syndrome [8,9], with infection rates reaching up to 90% [10]. Subsequent studies have shown that the virus can be transmitted through direct contact with infected birds, vertical transmission via eggs [11,12,13], contaminated environments, and possibly through vectors such as mosquitoes, raising concerns about its impact on poultry health and broader ecosystems [14]. Due to the virus’s high transmission capability, increased surveillance and biosecurity measures in poultry farming are essential to reduce the risk of outbreaks and protect animal and public health.

DTMUV is a positive-sense single-stranded RNA virus enclosed by a double-layered lipid envelope containing spike proteins. Its genome, spanning 11 kb, consists of one open reading frame (ORF) flanked by 5′ and 3′ untranslated regions (UTRs) [15]. The 5′ UTR served as the initiation site for positive-strand nucleic acid replication. The 3′ end lacks a poly(A) tail but displays a relatively conserved structure crucial for viral replication and transcription. The ORF encodes a 3425-amino acid polyprotein, which is post-translationally cleaved by host and viral proteases into three structural proteins (envelope (E), pre-membrane (prM), and capsid (C)) and seven non-structural proteins (NS1, NS2A, NS2B, NS3, NS4A, NS4B, and NS5) [16]. Structural proteins play roles in viral particle assembly, infection mediation, and immune response elicitation [17,18], while non-structural proteins drive viral replication and assembly, facilitating evasion of the host immune surveillance system [18,19].

Ducks and geese serve as primary hosts for DTMUV, which exhibits high pathogenicity towards these avian species, affecting various breeds and age categories. Chickens and pigeons may also contract the virus, although such occurrences are infrequent. In farms, infection rates can reach as high as 90%, while mortality rates can range from 5% to 30%, contingent on rearing conditions. Infected ducklings exhibit severe neurological disorders, including encephalitis, head and neck twisting, convulsions, head tilt, circling, and ataxia, along with ruffled feathers, emaciation, yellowish-green diarrhea, and elevated body temperature (42–43 °C) [20]. In laying ducks, primary clinical signs include a significant decrease in egg production and the laying of soft-shelled or thick-shelled eggs, with production rates plummeting from 90% to 10%. In severe cases, egg-laying ceases entirely [21]. Additionally, affected birds may display signs of respiratory distress, such as coughing and nasal discharge, further complicating their overall health. The rapid onset of these symptoms often results in high mortality rates within affected populations. Surviving individuals may suffer lasting effects on their neurological function and reproductive capabilities, impacting flock productivity and welfare in the long term. Pathological changes in DTMUV-infected laying ducks primarily involve extensive ovarian damage, accompanied by funicular hemorrhage, congestion, ovarian necrosis, and yolk peritonitis [22]. In male ducks, testicular atrophy is the primary manifestation. Secondary infections may result in hemorrhage, inflammation, hyperplasia, and macrophage/lymphocyte infiltration in the spleen, liver, kidneys, and brain [23].

In light of the cross-species characteristics of DTMUV and the substantial losses it has inflicted on the poultry sector in China, we analyzed the most recent epidemiological situation of DTMUV in the country. During this study, a total of 2674 avian swab samples were obtained from 48 sites across seven provinces in China in 2024. These samples were collected from various natural hosts of DTMUV, including chickens, ducks, geese, and pigeons, sourced from wholesale markets, slaughterhouses, farmers’ markets, and poultry farms. DTMUV was detected through qPCR techniques, and an analysis of the geographical, host, and site distribution of DTMUV was conducted to ascertain its epidemic status in China. Concurrently, positive samples underwent NS5 gene amplification, and the genetic evolutionary relationships were examined to offer insights for the comprehensive prevention and control of DTMUV.

## 2. Materials and Methods

### 2.1. Animal Ethics Statement

The Animal Ethics Committee of the Chinese Center for Animal Health and Epidemiology (DWFL-2023-07) approved this study (Approval Date: 20 October 2023). All procedures involving animals were conducted according to the guidelines for the care and use of experimental animals established by the Chinese Center for Animal Health and Epidemiology of China.

### 2.2. Clinical Samples and Cells

In 2024, a total of 2674 avian throat swab samples were collected from geese, chickens, ducks, and pigeons, with 1379 from chickens, 946 from ducks, 238 from geese, and 111 from pigeons. These samples were obtained from poultry farms, slaughterhouses, wholesale markets, and retail markets in Jiangsu, Anhui, Fujian, Henan, Guangdong, Guangxi, and Ningxia provinces. Further details are provided in Table 1. The swabs were immersed in 1 mL of phosphate-buffered saline (PBS) containing dual antibiotics. After thorough mixing using a vortex mixer (Sangon Biotech, Shanghai, China), the samples underwent centrifugation at 10,000 rpm for 5 min, following which the supernatant was harvested for subsequent analysis. BHK-21 cells were employed in the viral isolation of DTMUV. These cells were preserved in our laboratory, with their growth medium composed of Dulbecco’s modified Eagle’s medium (DMEM) supplemented with 10% fetal bovine serum (FBS) and their maintenance medium consisting of DMEM with 1% FBS.

### 2.3. Detection by Fluorescent Quantitative RT-PCR

To detect DTMUV in the original swab, RNAs were extracted following the provided instructions for the FineMag Rapid Magnetic Bead-Based Virus DNA/RNA Extraction Kit (GENFINE, Beijing, China). Magnetic beads were resuspended in Buffer MWP; then, 200 μL of sample supernatant and 20 μL of Proteinase K with Buffer MVN were mixed, and nucleic acid extraction was performed in Buffer DW1P with the stirrer sleeve. Subsequently, the fluorescent quantitative RT-PCR method was utilized to analyze 2674 avian throat swab samples collected from 7 provinces in China in 2024. Primer sequence Flavivirus-F: AGACTGCTGGTGCAATGAGAC; Flavivirus-R: GTGATGCACGTTCACCGATC; Flavivirus-P: FAM-AGACCTCCGTCCATGCCGTGAAGG-BHQ1. The reaction system consisted of 10 μL of 2× Pro Taq HS Probe Premix III (Vazyme, Nanjing, China), 6.8 μL of RNase-free water, 0.4 μL of Flavivirus-F, 0.4 μL of Flavivirus-R, 0.4 μL of Flavivirus-P, 2 μL of DNA template, and RNase-free water to make up a total volume of 20 μL. The reaction conditions involved incubation at 42 °C for 5 min, initial denaturation at 95 °C for 30 s, followed by denaturation at 95 °C for 5 s, annealing at 60 °C for 30 s, and 40 cycles.

### 2.4. Virus Isolation Using BHK-21

Due to the low viral RNA concentration in the original swab samples, direct amplification of the NS5 gene fragment was not feasible. Therefore, virus isolation in BHK-21 cells was performed. Positive swab samples from nucleic acid testing are to be added to the supernatant of cultured BHK-21 cells and incubated at 37 °C for 2 h. After discarding the cell growth solution, the cells were washed twice with sterile PBS, replaced with cell maintenance solution, and cultured for 5 days at 37 °C and 5% CO_2_. If no cytopathic effects were observed, the cells underwent three passages. If cytopathic effects were present, RNA was extracted from the supernatant for fluorescence quantitative RT-PCR to detect DTMUV infection, and the samples were stored at −80 °C for further analysis.

### 2.5. RT-PCR Amplification of the Partial DTMUV NS5 Gene

To obtain the partial DTMUV NS5 gene sequence, RT-PCR amplification was conducted using cell culture supernatant samples. The partial NS5 gene fragment amplified in this study is approximately 330 bp in length, corresponding to nucleotide positions 3172–3501 of the DTMUV genome (based on reference strain DTMUV/QY/17, GenBank: MT447092.1). This region encodes a conserved segment of the RNA-dependent RNA polymerase (RdRP) domain within the NS5 protein. The RT-PCR amplification system (total volume 50 μL) comprised 1 μL each of upstream DTMUV-F: GAGTGAACTCATCATACC and downstream primers DTMUV-R: GATTTGATCAACATGTCGTCT, 25 μL of 2× Mix (Dye Plus), 5 μL of RNA template, and 18 μL of ddH_2_O. The RT-PCR amplification procedure involved reverse transcription at 50 °C for 30 min, pre-denaturation at 95 °C for 5 min, denaturation at 94 °C for 30 s, annealing at 58 °C for 45 s, and extension at 72 °C for 60 s for 35 cycles. Subsequently, the samples underwent sequencing by Beijing Qingke Biotechnology Co., Ltd. (Qingdao, China).

### 2.6. Sequence Analysis

DNAStar (v7.1) software was utilized to assemble sequences for the generation of viral NS5 gene sequences. A total of 17 samples with high-quality sequencing results were acquired. The information regarding these strains is presented in Table 2. MegAlign (v7.1) was utilized to conduct nucleotide sequence homology analysis comparing the obtained DTMUV NS5 gene sequence with 26 DTMUV gene sequences from GenBank, encompassing both domestic and international strains (Table 2). Subsequently, 17 sequences were subjected to multiple sequence alignment. A phylogenetic tree was constructed using MEGA11 software employing the neighbor-joining method with 1000 bootstrap replicates to examine the genetic evolutionary characteristics of the virus.

## 3. Results

### 3.1. Source Distribution of DTMUV

**Figure 1:**
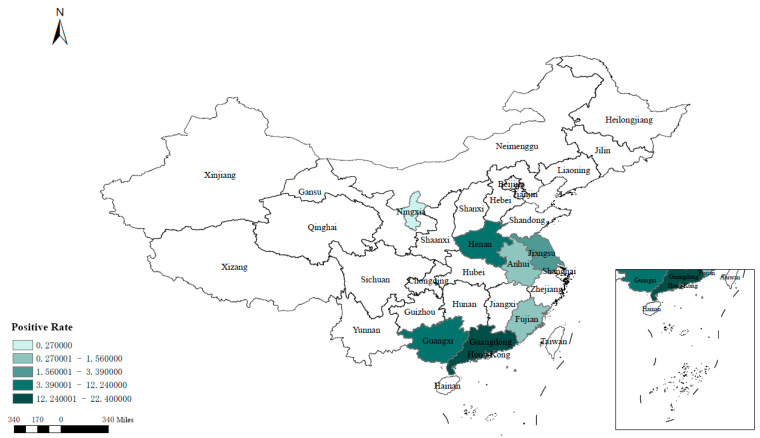
Regional distribution of DTMUV.

Fluorescent quantitative RT-PCR testing was performed on 2674 swab samples collected from seven provinces in China, revealing the presence of DTMUV in all surveyed regions. Among the samples, 198 tested positive, resulting in an overall positivity rate of 7.40% (198/2674). Analysis depicted in Figure 1 indicated higher positivity rates in the southern and central provinces, notably Guangdong (22.40%, 86/384) and Henan (12.24%, 47/384), while the eastern and northwestern provinces exhibited lower rates, such as Anhui (1.04%, 4/384) and Ningxia (0.27%, 1/370).

### 3.2. Host Distribution of DTMUV

Evidence of cross-host transmission of DTMUV is apparent from the findings presented in Table 1, RT-qPCR results. with positive cases detected in chickens, ducks, geese, and pigeons, albeit at varying rates. Geese display the highest positivity rate at 40.76% (97/238), while chickens and ducks show rates of 4.44% (57/1379) and 4.13% (42/946), respectively. Pigeons can also contract DTMUV, albeit with a lower positivity rate of 1.80% (2/111).

### 3.3. Distribution of DTMUV Sampling Sites

Sampling locations in the epidemiological investigation of this study were diversified. According to Table 3, DTMUV-positive samples were detected in 31 sites, encompassing 6 wholesale markets, 3 slaughterhouses, 10 farmers’ markets, and 12 poultry farms. The overall positivity rate across all locations was 64.58% (31/48). Positivity rates of samples collected from various sampling locations were also assessed. The findings indicated that samples from poultry farms exhibited the highest positivity rate at 16.00% (101/630), followed by slaughterhouses at 7.50% (25/330). The positivity rates for samples from wholesale markets and farmers’ markets were 4.10% and 4.20%, respectively (Table 3).

### 3.4. Homology Analysis of Partial NS5 Gene of DTMUV

Following the inoculation of BHK-21 cells with the throat swab samples, cytopathic effects were monitored, and cell supernatants were collected for further analysis. Subsequently, 17 viral strains were isolated, and sequencing was performed on partial NS5 gene sequences of these isolates. The 17 sequenced strains originated from three provinces (Guangdong, Henan, and Guangxi) and three host species (geese, chickens, and ducks), representing a subset of the positive samples. Comparative analysis was conducted between these NS5 gene fragments and DTMUV NS5 gene sequences accessible in the GenBank database to determine sequence homology. The results indicated nucleotide sequence homologies ranging from 80.3% to 99.4% between the 17 isolates and the endemic DTMUV reference strain.

### 3.5. Genetic Evolution Analysis of the Partial Zai Gene of DTMUV

**Figure 2:**
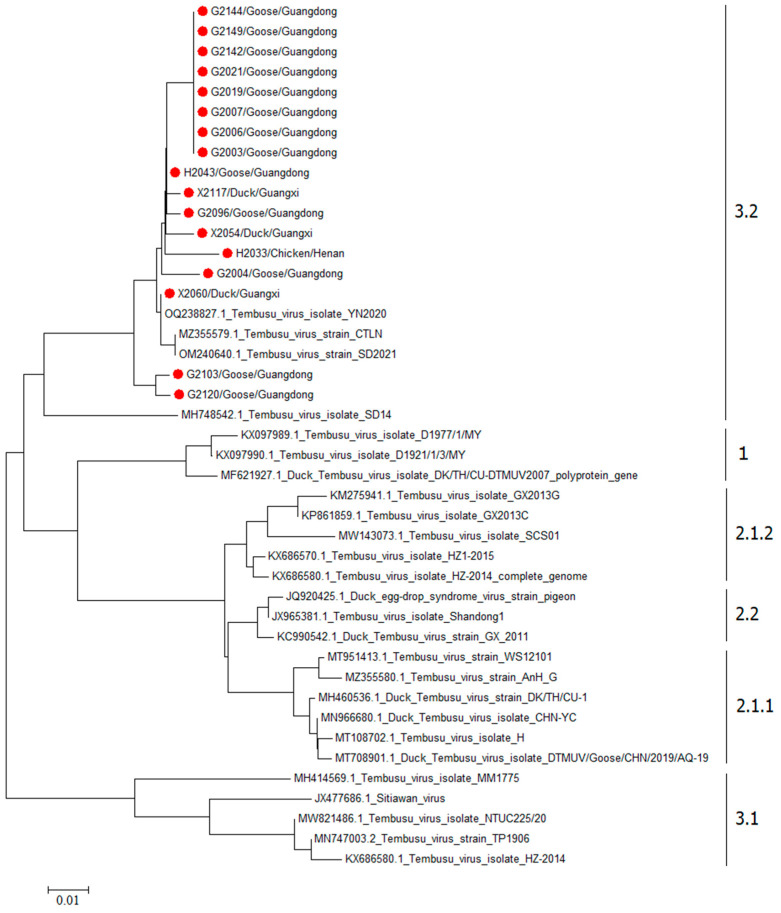
Neighbor-joining tree based on partial NS5 gene sequences of DTMUV.

Phylogenetic analysis was conducted using the neighbor-joining method by MEGA11 on 17 DTMUV strains and 26 reference sequences sourced from NCBI to construct a phylogenetic tree. The analysis revealed distinct epidemiological lineages within DTMUV based on genetic evolutionary relationships. Clade 1 primarily consisted of strains isolated from ducks in Malaysia. Clade 2 was further divided into subclades 2.1 and 2.2, predominantly comprising Thai and Chinese DTMUV isolates. Chinese isolates predating 2013 clustered within subclade 2.2, while more recent isolates clustered within subclade 2.1. Cluster 3 encompassed subclades 3.1 and 3.2, with DTMUV isolates in subclade 3.2 demonstrating an increasing prevalence in recent years. All 17 DTMUV isolates from this study were classified under subclade 3.2 in the phylogenetic tree (Figure 2).

## 4. Discussion

Since the first documented major outbreak of DTMUV in duck flocks in China in 2010 [6], the virus has re-emerged as a significant pathogen affecting poultry production across multiple countries in Southeast Asia. In China, the initial outbreaks in 2010–2011 were characterized by sudden egg-drop syndrome in laying ducks, with infection rates reaching up to 90% and economic losses [10]. Following these outbreaks, DTMUV rapidly spread to neighboring provinces and was subsequently reported in Thailand [24], Malaysia [25], and Vietnam [26], establishing endemic circulation throughout the region. The virus demonstrated remarkable genetic diversity during this expansion, with phylogenetic analyses revealing the emergence of multiple clades and subclades across different geographic regions and host species. Notably, DTMUV outbreaks have continued to occur intermittently in China and Southeast Asia throughout the 2010s and into the 2020s, with shifting predominant genotypes and expanding host ranges [27]. This study was therefore conducted to assess the current epidemiological status of DTMUV in China in 2024 and to characterize the genetic features of circulating strains.

In this study, a comprehensive survey was conducted across different provinces and species in China to assess the prevalence of DTMUV infections among diverse hosts. DTMUV-positive samples were detected in all seven provinces in China, with a provincial positivity rate of 7.40%. Positivity rates varied across regions, ranging from 0.27% to 22.40%. The disparity in rates could be attributed to the humid and hot climates in the southern regions (e.g., Guangdong, Guangxi), which favor mosquito breeding and enhance viral transmission. In contrast, the northwest region (e.g., Ningxia) experiences a dry climate and low mosquito abundance, leading to significantly lower positivity rates. These findings suggest that while DTMUV infection rates are relatively high in China, there are distinct regional variations in their distribution. Higher positivity rates were observed in poultry farms and slaughterhouses, possibly due to high stocking densities, poor environmental hygiene, and inadequate disinfection practices. Wholesale markets and slaughterhouses had higher positivity rates compared to farmers’ markets and poultry farms, likely attributed to increased personnel and vehicle movement at wholesale markets, facilitating virus introduction. Slaughterhouses, serving as convergence points for multiple poultry batches, support sustained viral circulation. These results underscore the widespread prevalence and high transmissibility of DTMUV in China.

It is known that DTMUV demonstrates a notable ability for interspecies transmission, affecting ducks, geese, chickens, sparrows, and various wild birds. It is noteworthy that DTMUV can induce fatal encephalitis and systemic infection in BALB/c and Kunming mice through intracerebral inoculation, underscoring its relevance to public health. We focused on analyzing the infection rates of DTMUV in different hosts. The results revealed that geese had the highest infection rate at 40.76% (97/238), while ducks and chickens had infection rates around 4%. Although ducks are commonly considered the primary host for DTMUV, their widespread vaccination against the virus may account for their relatively lower infection rates. Geese, on the other hand, are generally not vaccinated against DTMUV and may be more susceptible to the virus, potentially facilitating its maintenance through vector transmission in goose populations. Studies suggest that the higher infection rate in geese, compared to ducks, may be due to geese’s lack of effective immune response to DTMUV, while certain duck breeds exhibit partial genetic resistance. The high infection rate in geese is concerning, especially in China, where waterfowl (ducks and geese) often share water sources. Despite geese showing mild symptoms when infected with DTMUV, they can transmit the virus to ducks and other animals, leading to a broader infection spread. Furthermore, despite the low infection rate among pigeons, continued monitoring is warranted. Given the wide-ranging movements of pigeons, they could serve as significant vectors for transmission, underscoring the importance of implementing effective biosecurity measures against birds in waterfowl farms.

In this study, we further isolated DTMUV using BHK-21 cells. Among 198 RT-qPCR-positive samples, we isolated 17 strains of the virus. The low isolation rate may be attributed to low viral loads in swab samples and potential virus inactivation during transportation and storage under variable field conditions. As a single-stranded positive-sense RNA virus, DTMUV is susceptible to nucleotide mutations, potentially leading to changes in amino acid composition, antigenicity, and pathogenicity [28]. The NS5 protein, the largest protein encoded by DTMUV, exhibits a highly conserved nucleic acid sequence critical for viral genome replication and immune evasion [29]. Comparative analysis of NS5 gene fragments from 17 selected DTMUV isolates and domestic representative strains showed homology levels ranging from 80.3% to 99.4%. Phylogenetic analysis confirmed that all isolates in the study belong to the 3.2 subgroup. The high homology and genetic evolutionary relationships suggest that cross-species infections may play a significant role in DTMUV transmission. This indicates a gradual increase in the prevalence of the 3.2 subgroup of DTMUV in China, potentially becoming the predominant circulating subgroup nationally.

Beyond the predominant subclade 3.2 identified in this study, DTMUV strains circulating in China exhibit considerable genetic diversity, with three major clusters (1, 2, and 3) [30,31]. Historically, Cluster 2.2 was the dominant lineage associated with the initial duck outbreaks in 2010–2013, causing significant economic losses in major duck-producing provinces in China [30]. This clade continues to circulate in duck populations, although its prevalence has declined in recent years, possibly due to widespread vaccination programs targeting Cluster 2 strains. In contrast, Cluster 3, particularly subclade 3.2, has emerged as an increasingly important lineage since approximately 2019 [32]. Unlike Cluster 2 strains, Cluster 3.2 exhibits broader host tropism with infections in geese, chickens, and pigeons across multiple provinces in China. Studies have shown that some chicken-origin Cluster 3.2 strains display higher infectivity in chicks compared to traditional duck-origin Cluster 2 strains, suggesting enhanced adaptation to terrestrial poultry. The co-circulation of multiple genetic clades in Chinese poultry populations underscores the complex evolutionary dynamics of DTMUV and highlights the need for continued genomic surveillance. The predominance of subclade 3.2 in our study aligns with the national trend toward increasing Cluster 3.2 prevalence, although we acknowledge that our sampling was limited to seven provinces and may not fully represent the genetic diversity in other regions. Future studies should include broader geographic sampling to better understand the evolutionary trajectories and functional significance of genetic variations among DTMUV clades circulating in China.

It is important to note that several limitations of this study need to be acknowledged. First, the 17 sequenced strains are not fully representative of all 198 positive samples, as they originated from only three provinces and three host species. Second, all isolates belonged to subcluster 3.2, which may reflect either the true predominance of this lineage or a sampling bias. Third, the low virus isolation rate highlights the challenges of field-sample-based surveillance for RNA viruses. Future studies should aim for larger-scale sequencing across more regions and host species to better understand the genetic diversity and transmission dynamics of DTMUV in China.

In summary, an epidemiological survey of DTMUV was carried out across seven provinces in China in this study, demonstrating host diversity among DTMUV strains. The heightened infection rate observed in geese emphasizes the critical significance of investigating their contribution to DTMUV transmission. Consequently, the effective prevention and control of DTMUV in China are hindered by substantial challenges, emphasizing the essential role of reinforcing biosecurity measures in poultry farms.

## Figures and Tables

**Table 1:** Sample collection quantities from different provinces.

Province	Chickens			Ducks			Geese			Pigeons		
	Total	RT-qPCR	Virus Isolation	Total	RT-qPCR	Virus Isolation	Total	RT-qPCR	Virus Isolation	Total	RT-qPCR	Virus Isolation
Jiangsu	204	9	0	125	4	0	0	0	0	55	0	0
Anhui	244	4	0	137	0	0	0	0	0	3	0	0
Fujian	189	3	0	194	2	0	1	1	0	0	0	0
Henan	184	32	2	180	13	0	15	1	0	5	1	0
Guangdong	164	2	0	70	3	0	130	81	12	20	0	0
Guangxi	81	6	0	209	20	3	69	14	0	25	1	0
Ningxia	313	1	0	31	0	0	24	0	0	2	0	0
Total	1379	57	2	946	42	3	238	97	12	111	2	0

Note: RT-qPCR and virus isolation represent the number of positive samples.

**Table 2:** The 17 isolated strains and reference sequence information.

Name	Host	Country	Year	Location	GeneBank
G2003	Goose	China: Guangdong	2024	Poultry farm	PX421573
G2004	Goose	China: Guangdong	2024	Poultry farm	PX453337
G2006	Goose	China: Guangdong	2024	Poultry farm	PX453338
G2007	Goose	China: Guangdong	2024	Poultry farm	PX453339
G2019	Goose	China: Guangdong	2024	Poultry farm	PX453340
G2021	Goose	China: Guangdong	2024	Poultry farm	PX453341
G2096	Goose	China: Guangdong	2024	Poultry farm	PX453342
G2103	Goose	China: Guangdong	2024	Poultry farm	PX453343
G2120	Goose	China: Guangdong	2024	Poultry farm	PX453344
G2142	Goose	China: Guangdong	2024	Poultry farm	PX453345
G2144	Goose	China: Guangdong	2024	Poultry farm	PX453346
G2149	Goose	China: Guangdong	2024	Poultry farm	PX453347
H2033	Chicken	China: Henan	2024	Slaughterhouse	PX453348
H2043	Chicken	China: Henan	2024	Slaughterhouse	PX453349
X2054	Duck	China: Guangxi	2024	Poultry farm	PX453350
X2060	Duck	China: Guangxi	2024	Poultry farm	PX453351
X2117	Duck	China: Guangxi	2024	Wholesale Market	PX453352
DK/TH/CU-DTMUV2007	Duck	Thailand	2007	-	MF621927
Tembusu virus isolate D1977/1/MY	Duck	Malaysia	2012	-	KX097989
Tembusu virus isolate D1921/1/3/MY	Duck	Malaysia	2012	-	KX097990
Duck Tembusu virus isolate CHN-YC	Duck	China	2019	-	MN966680
DTMUV/Goose/CHN/2019/AQ-19	Goose	China	2019	-	MT708901
DK/TH/CU-1	mosquito	Thailand	2018	-	MH460536
Tembusu virus strain AnH G	Goose	China	2021	-	MZ355580
Tembusu virus strain WS12101	Goose	China	2020	-	MT951413
Tembusu virus isolate H	Duck	China	2020	-	MT108702
Tembusu virus isolate HZ1-2015	Duck	China	2016	-	KX686570
Tembusu virus isolate SCS01	Duck	China	2020	-	MW143073
Tembusu virus isolate HZ-2014	Duck	China	2016	-	KX686580
Tembusu virus isolate GX2013C	Duck	China	2015	-	KP861859
Tembusu virus isolate GX2013G	Duck	China	2014	-	KM275941
Duck Tembusu virus strain GX_2011	Duck	China	2013	-	KC990542
Tembusu virus isolate Shandong1	Duck	China	2012	-	JX965381
Duck egg-drop syndrome virus strain pigeon	Pigeon	China	2012	-	JQ920425
Tembusu virus isolate NTUC225/20	Wild Geese	China	2021	-	MW821486
Tembusu virus strain TP1906	mosquito	China	2019	-	MN747003
Tembusu virus isolate 1080905	Mallard	China	2021	-	MW922032
Sitiawan virus	mosquito	Malaysia	2012	-	JX477686
Tembusu virus isolate MM1775	mosquito	Malaysia	2018	-	MH414569
Tembusu virus strain CTLN	Chicken	China	2012	-	MZ355579
Tembusu virus isolate SD14	Mallard	China	2018	-	MH748542
Tembusu virus isolate YN2020	mosquito	China	2023	-	OQ238827
Tembusu virus strain SD2021	Chicken	China	2022	-	OM240640

Note: The first 17 strains are the isolates from this study, while the remaining strains provide reference sequence information.

**Table 3:** Distribution of DTMUV field points and field point samples.

Type	Number of Sites	Number of Positive Sites	Positive Rate	Number of Samples	Number of Positive Samples	Positive Rate
Wholesale Market	8	6	75%	942	39	4.10%
Slaughterhouse	4	3	75%	330	25	7.50%
Farmers’ Market	15	10	66.67%	772	33	4.20%
Poultry Farm	21	12	57.14%	630	101	16.00%
Total	48	31	64.58%	2674	198	7.4%

## Data Availability

The data that support the findings of this study are available from the corresponding author upon reasonable request.
